# Deciphering the molecular regulatory of RAB32/GPRC5A axis in chronic obstructive pulmonary disease

**DOI:** 10.1186/s12931-024-02724-2

**Published:** 2024-03-06

**Authors:** Yixing Wu, Binfeng He, Jianlan Hua, Weiping Hu, Yaopin Han, Jing Zhang

**Affiliations:** 1 Department of Pulmonary and Critical Care Medicine, Zhongshan Hospital, Fudan University, Shanghai, China; 2 Department of General Practice, Xinqiao Hospital, Third Military Medical University, Chongqing, China

**Keywords:** Chronic obstructive pulmonary disease, RAB32, GPRC5A, WGCNA, Single-cell RNA sequencing

## Abstract

**Background:**

Chronic obstructive pulmonary disease (COPD) is a significant public health problem characterized by persistent airflow limitation. Despite previous research into the pathogenesis of COPD, a comprehensive understanding of the cell-type-specific mechanisms in COPD remains lacking. Recent studies have implicated Rab GTPases in regulating chronic immune response and inflammation via multiple pathways. In this study, the molecular regulating mechanism of RAB32 in COPD was investigated by multiple bioinformatics mining and experimental verification.

**Methods:**

We collected lung tissue surgical specimens from Zhongshan Hospital, Fudan University, and RT-qPCR and western blotting were used to detect the expression of Rabs in COPD lung tissues. Four COPD microarray datasets from the Gene Expression Omnibus (GEO) were analyzed. COPD-related epithelial cell scRNA-seq data was obtained from the GSE173896 dataset. Weighted gene co-expression network analysis (WGCNA), mfuzz cluster, and Spearman correlation analysis were combined to obtain the regulatory network of RAB32 in COPD. The slingshot algorithm was used to identify the regulatory molecule, and the co-localization of RAB32 and GPRC5A was observed with immunofluorescence.

**Results:**

WGCNA identified 771 key module genes significantly associated with the occurrence of COPD, including five Rab genes. RAB32 was up-regulated in lung tissues from subjects with COPD as contrast to those without COPD on both mRNA and protein levels. Integrating the results of WGCNA, Mfuzz clusters, and Spearman analysis, nine potential interacting genes with RAB32 were identified. Among these genes, GPRC5A exhibited a similar molecular expression pattern to RAB32. Co-expression density analysis at the cell level demonstrated that the co-expression density of RAB32 and GPRC5A was higher in type I alveolar epithelial cells (AT1s) than in type II alveolar epithelial cells (AT2s). The immunofluorescence also confirmed the co-localization of RAB32 and GPRC5A, and the Pearson correlation analysis found the relationship between RAB32 and GPRC5A was significantly stronger in the COPD lungs (*r* = 0.65) compared to the non-COPD lungs (*r* = 0.33).

**Conclusions:**

Our study marked endeavor to delineate the molecular regulatory axis of RAB32 in COPD by employing diverse methods and identifying GPRC5A as a potential interacting molecule with RAB32. These findings offered novel perspectives on the mechanism of COPD.

**Supplementary Information:**

The online version contains supplementary material available at 10.1186/s12931-024-02724-2.

## Introduction

Chronic obstructive pulmonary disease (COPD) is a complex, heterogeneous lung disease characterized by persistent airflow limitation with chronic inflammation [1, 2]. With 10% of the global population affected, COPD is a significant public health problem [3]. In accordance with the World Health Organization, COPD-related deaths are rising consistently, even predicted to become the third leading cause of death worldwide by 2030 [4]. Comprehensive transcriptome analysis and genome-wide association studies (GWAS) using integrated mouse models or large human lung tissue datasets have provided a greater understanding of the mechanisms underlying the pathogenesis of COPD in previous research [5–9]. Several biological processes have been identified as being activated in the pathogenesis of COPD, including chronic immune response and inflammation, cellular metabolic dysfunction, DNA damage, cell apoptosis and autophagy, and protease/antiprotease imbalance pathways [5, 10–12]. However, a comprehensive understanding of the interplay between cell-type-specific mechanisms and multi-hierarchical regulatory systems in COPD is still lacking.

Ras-associated binding (Rab) GTPases, a small GTPase family within the Ras superfamily, have been implicated in the regulation of chronic immune response and inflammation via multiple pathways in recent years [13–15]. Following LPS treatment of macrophages, upregulation of the expression of RAB1 [16], RAB10 [17], and RAB21 [18] can induce the production of large quantities of inflammatory cytokines, resulting in sustained lung tissue injury. Our previous study also found that down-regulation of RAB26 increased the p38 and JNK-MAPK signaling pathways, thereby exacerbating cigarette smoke extract-induced inflammatory responses [19]. Therefore, we attempted to elucidate the cell-type-specific potential mechanisms of Rabs in COPD at the “molecular-cellular” levels by integrating transcriptomics, single-cell RNA sequencing (scRNA-seq), spatial transcriptomics, and biological experiments to better understand the pathobiology of this disease.

In this study, we examined the transcriptional characteristics of COPD to identify its specific regulatory mechanisms using weighted gene co-expression network analysis (WGCNA). With WGCNA and quantitative analysis of lung tissues from patients with solitary pulmonary nodules, RAB32, which might regulate the pathological mechanisms of COPD, was identified. Soft clustering Mfuzz patterns were used to identify molecular modules that were consistent with RAB32 expression characteristics, and the biological functions of RAB32 in COPD were uncovered utilizing enrichment analysis. ScRNA-seq pseudotime analysis was applied to identify potential molecules interacting with RAB32 in alveolar epithelial cells with similar temporal expression characteristics to RAB32, and biological experiments were used to further validate. Besides, the correlation between RAB32 and other molecules were analyzed based on the clinical cohort. According to our knowledge, this is the first endeavor using multiple methods to reveal the molecular regulatory axis of RAB32 in COPD, which may provide new insights into the diagnosis and treatment of the disease.

## Methods and materials

The overall study design is shown in Fig. S1.

### Patients and samples

Patients with operable, solitary lung nodules were recruited from Zhongshan Hospital, Fudan University, Shanghai, China. Patients diagnosed as SCLC or lung squamous cell carcinoma, and parients with other chronic lung diseases were excluded. Then patients were divided into COPD and non-COPD group according to the diagnosis of COPD in GOLD (Global initiative for chronic obstructive lung disease) guideline criteria. Their demographic characteristics were collected, and normal lung tissue samples were obtained from distal resection margins (> 2 cm away from the lung nodule) after lobectomy surgery for experiments and quantitative analysis. The study was approved by the Ethics Committee of Zhongshan Hospital of Fudan University (B2018-137R) and all the participants have signed an informed consent.

### Western blotting

The human lung tissue samples were lysed with RIPA lysis buffer (Beyotime Biotechnology, China, #P0013) and protease inhibitor cocktail (Beyotime Biotechnology, China, #P1005). Then the total protein was separated by SDS-PAGE and transferred to a PVDF membrane. Following blocking in 5% skim milk, the membrane was incubated with primary antibodies overnight and the horseradish peroxidase-conjugated secondary antibody for one hour at room temperature. The bands were detected in the *CLINX chemiluminescence imaging system* and quantified using the Image J software (version 2.1.0). All target protein was normalized using GAPDH and the target protein in the control group was considered as the baseline (value = 1). Antibodies were as follows: GAPDH antibody (Cell Signaling Technology, USA, #5174), RAB32 Antibody (Thermo Fisher Scientific, Waltham, USA, #PA5-104073), GPRC5A Antibody (Abmart, Wuhan, China, #TD5148), Goat Anti-Rabbit IgG-HRP (Abmart, Wuhan, China, #M21002).

### Real-time quantitative PCR (RT-qPCR)

Total RNA was isolated from human lung tissues using RNAiso Plus regents (Takara, Beijing, China, #9108) and RNA simple isolation kit (Tiangen, Beijing, China, #DP419). The PrimeScript™ RT reagent Kit with gDNA Eraser (Takara, Beijing, China, #RR047A) was used to perform reverse transcription to synthesize cDNAs. The RT-qPCR was performed using the *QuantStudio 5*, and the relative expression of target genes was normalized to β-actin. The primer sequences were as follows: β-actin forward, 5′-CATGTACGTTGCTATCCAGGC-3′, reverse, 5′-CTCCTTAATGTCACGCACGAT-3’. RAB6B forward, 5′-TGTACGACAGCTTCGACAACA-3′, reverse, 5′-CTGCGGAACCTCTCCTGAC-3’. RAB13 forward, 5′-TTGCAGAGGACAACTTCAACAA-3′, reverse, 5′-CTATATCCACAGTGCGGATCTTG-3’. RAB32 forward, 5′-CAGGTGGACCAATTCTGCAAA-3′, reverse, 5′-GGCAGCTTCCTCTATGTTTATGT-3’. RAB38 forward, 5′-GGGGAAGACCAGTATCATCAAGC-3′, reverse, 5′-CGGTAATAGACCCTCGTCATGT − 3’. RAB40B forward, 5′-GTCCGGGCCTACGACTTTC-3′, reverse, 5′-GGCCTGAAGTATCCCAGAGC-3’.

### Cell culture and immunofluorescence

A549 cell line was purchased from ATCC and cultured with Dulbecco’s Modified Eagle Medium (KeyGEN Biotech, Nanjing, China, #KGM12800) and 10% fetal bovine serum (Gibco, USA, #10,099,141 C) at 37 °C with 5% CO_2_. Forty-eight hours after transfection of the plasmid encoding RAB32-GFP, the cells were fixed with 4% paraformaldehyde and then permeabilized with 0.1% Triton X-100 (Sigma-Aldrich, USA, #T9284). Then they were stained with the primary antibody (Cell Signaling Technology, USA, #12,968) and appropriate fluorescence-conjugated secondary antibody (Abmart, Wuhan, China, #M21014). Confocal images were recorded using the *Olympus FluoView FV3000* confocal microscope.

### Immunohistochemistry

The fresh tumor tissues were fixed with 4% paraformaldehyde and embedded in paraffin, then cut into 5 μm sections. After dewaxing, rehydration, antigen repair, and blocking, the slides were incubated with primary antibodies (RAB32: Thermo Fisher Scientific, Waltham, USA, #PA5-104073, GPRC5A: Cell Signaling Technology, USA, #12,968) overnight. The slides were incubated with horseradish peroxidase-labelled secondary antibodies (Zhongshan Golden Bridge, Beijing, China, #PV-9000) the next day. DAB was used to visualize the reaction, and hematoxylin was used to label the nuclei. The histological score (H-score) was calculated using the following formula: H-SCORE = ∑(pi × i) = (percentage of weak intensity × 1) + (percentage of moderate intensity × 2) + (percentage of strong intensity × 3).

### Data retrieval and processing

We downloaded four mRNA microarray datasets (GSE5058, GSE10006, GSE11784, and GSE20257) [20–23] from the Gene Expression Omnibus database (GEO, https://www.ncbi.nlm.nih.gov/geo/) on the same GEO platform (GPL570) using the GEOquery package in R. The raw data were preprocessed in R using the Robust Multi-array Average (RMA) algorithm for background correction and normalization. We additionally corrected for potential batch effects using the ComBat algorithm in the sva package [24]. Moreover, scRNA-seq data related to COPD were obtained from the GSE173896 dataset [25], and epithelial cell data were extracted for subsequent analysis.

### Weighted gene co-expression network construction

We used the WGCNA package [26] to perform weighted gene co-expression network analysis to identify genes associated with COPD development. The similarity matrix was characterized by Pearson correlation values and transformed into an adjacency matrix based on the weighted coefficients ß. The adjacency matrix was then converted into a topological overlap matrix (TOM), and different modules were identified using the dynamic tree-cutting method, with a minimum module size threshold of 50. We used Pearson correlation analysis to investigate the relationship between modules and disease features (COPD or non-COPD) to determine their relevance. Modules with *p*-value < 0.05 were considered significantly associated with clinical features, and the module with the highest correlation coefficient was designated as the central module. Furthermore, Venn diagrams generated using the VennDiagram package were used to identify intersection genes among the RAB32-related gene set, Mfuzz feature patterns, and WGCNA central module, which were considered potential interacting genes with RAB32 at the tissue level.

#### Mfuzz expression pattern clustering and RAB32

Expression-Related Features We used the “Mfuzz” package [27, 28] to cluster Mfuzz expression patterns based on RAB32 expression levels, and calculated the single-sample gene set enrichment analysis (ssGSEA) scores of different clustering modules to characterize their levels in the COPD and non-COPD groups. Then, we separately calculated the Spearman correlation between the clustering modules and RAB32 in the COPD and non-COPD groups, and selected the clustering modules that differed between the two groups. Finally, we obtained the gene module most closely related to COPD and RAB32 by analyzing these differential modules.

### Functional and pathway enrichment analyses of RAB32 in COPD

First, we performed a “Spearman” correlation analysis to identify gene sets that are significantly correlated with RAB32 in COPD using a cutoff of *p*-value < 0.05 and correlation coefficient > 0.3 (or < -0.3). By using Gene Ontology (GO) and Kyoto Encyclopedia of Genes and Genomes (KEGG) enrichment analyses, we studied the biological processes (BP), molecular functions (MF), cellular components (CC), and potential signaling pathways of the RAB32-related gene set, and analyzed them using the clusterProfiler package [29]. Disease Ontology (DO, http://disease-ontology.org) [30] and Disease Gene Network (DisGeNET, http://www.disgenet.org) [31] were identified using the DOSE package [32]. Finally, we used the Gene Set Variation Analysis (GSVA) R package [33] to perform Gene Set Enrichment Analysis (GSEA) on the gene expression matrix. Analyses with *p*-values < 0.05 were considered statistically significant.

### Regulatory network of RAB32 expression-related patterns

We extracted functional network and gene connectivity data using the STRING database (https://string-db.org/) [34]. STRING provides gene connectivity data based on several types of evidence (direct interaction, co-localization, gene regulation, and co-citation), grouping closely related genes together with high confidence (interaction score > 0.7). We then used the iGraph package to analyze the connectivity data from STRING and construct a protein-protein interaction (PPI) network, analyzing the PPI using betweenness to obtain network hub genes. At the same time, we analyzed the extracted connectivity data using edge-betweenness and random walk methods to highlight subnetworks or neighborhoods. We conducted an enrichment analysis of relevant biological/pathway terms on the obtained subnetworks using the STRING database [35]. Gene set enrichment analysis for disease terms was conducted using the clusterProfiler [29].

### Computational analysis of single-cell RNA sequencing data

We combined the single-cell gene expression data from all patient epithelial cells and normalized the data using SCTransform in Seurat [36]. The top 3000 highly variable genes (HVGs) were identified and used to stabilize UMI counts. Principal Component Analysis (PCA) was performed using HVGs, and a shared nearest neighbor graph and uniform manifold approximation and projection (UMAP) were constructed using the Louvain algorithm with the top 30 principal components and clustering units. After removing unstable clusters, HVGs were identified again and clustered using the Louvain algorithm. Based on cross-cluster typical cell type marker scores, we determined the major epithelial cell types: AGER, CLIC5, and PDPN were used to label type I alveolar epithelial cells (AT1s), and SFTPA1, SFTPA2, SFTPB, and SFTPC were used to label type II alveolar epithelial cells (AT2s).

### Trajectory inference analysis

The Slingshot algorithm was used to define a computational estimated pseudotime trajectory in alveolar epithelial cells. For each analysis, differential genes for each phenotype were reduced based on PCA and visualized in two dimensions using UMAP. The UMAP matrix was then fed into Slingshot to calculate the trajectory and pseudotime. These results were used to compute the trajectory using TradeSeq. We further adjusted the concept of TradeSeq to construct gene expression changes of RAB32 and key genes identified at the tissue level within the same trajectory to identify feature genes with similar expression to RAB32 in alveolar epithelial cells [37].

### Statistical analysis

Statistical analysis and graphics were performed and obtained using R software (version 4.2.0) and GraphPad Prism (version 8.0). Data were presented as mean ± standard deviation. Two groups were compared using Welch’s t-test or the Mann–Whitney U test (both two-sided), as indicated, after testing for normal distribution using the Shapiro–Wilk test. For the correlation analysis, the Spearman’s or Pearson’s correlation coefficient was calculated. A *p*-value < 0.05 was considered statistically significant.

## Results

### Gene co-expression network construction and identification of hub rabs

**Fig. 1:**
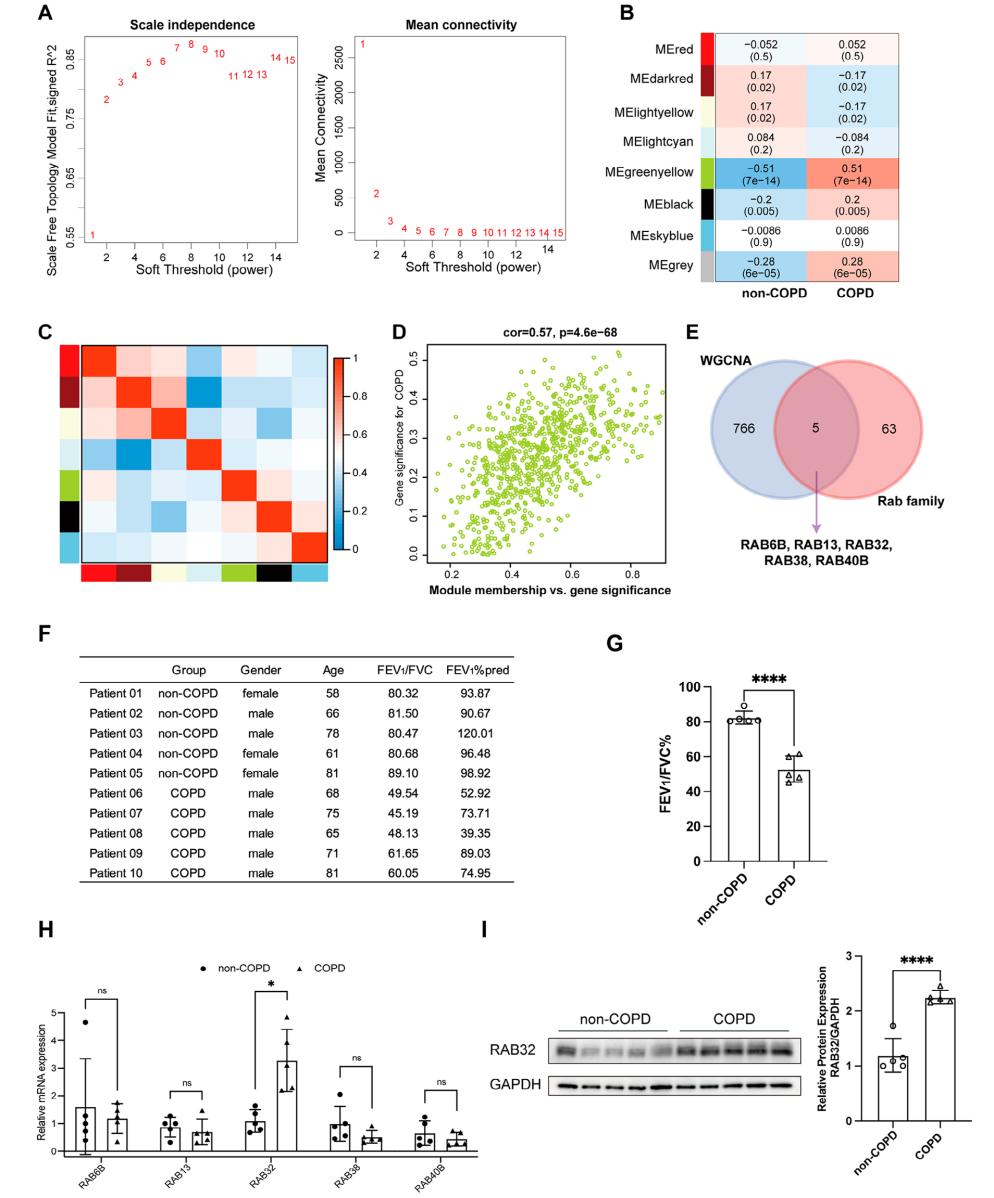
Identification of key Rab genes associated with COPD pathogenesis using WGCNA. (**A**) Evaluation of scale-free fit index and mean connectivity at different soft threshold powers. (**B**) Heatmap illustrating the correlation between module eigengenes and COPD status. Each cell displays the respective correlation coefficient and *P*-value. (**C**) Inter-correlation among WGCNA module eigengenes. (**D**) Scatter plot depicting the correlation between module membership within the MEgreenyellow module and Gene significance. (**E**) Venn diagram highlighting the intersection between WGCNA hub genes and the Rab GTPase family. (**F**) Clinical pathological parameters of ten lung tissues samples. (**G**) Bar plot demonstrating the difference in FEV_1_/FVC% between non-COPD and COPD groups (*n* = 5). (**H**) RT-qPCR analysis revealing mRNA expression differences of hub members within the Rab GTPase family between non-COPD and COPD groups (*n* = 5). (**I**) Western blotting and its quantification of RAB32 protein levels in lung tissues of non-COPD and COPD groups (*n* = 5). Note: ns denotes non-significance, * *P* < 0.05, ** *P* < 0.01, *** *P* < 0.001, **** *P* < 0.0001

Four publicly available COPD microarray datasets (GSE5058, GSE10006, GSE11784, GSE20257) from the GEO database were obtained for further analysis. These datasets were generated on the GPL570 platform, and thus, the batch effects were removed using the SVA package to conduct a comprehensive combined analysis. After excluding samples with ambiguous diagnoses or duplicates, a total of 30 COPD patients and 163 non-COPD control subjects participated in this study. The intricate regulatory processes underlying COPD occurrence were explored using the WGCNA approach. To construct a scale-free network, a soft threshold of 6 was applied (Fig. 1A). Gene modules were identified using TOM detection, resulting in the identification of eight modules. Analysis of the association between gene modules and COPD characteristics revealed that the MEgreenyellow module exhibited the strongest correlation with COPD (*r* = 0.51, *p* < 0.001, Fig. 1B). Apart from the MEgreenyellow module, only the MEblack module showed a weak correlation with COPD (*r* = 0.2, *p* = 0.005). Further characterization of the gene modules demonstrated the similarity between MEgreenyellow and MEblack (Fig. 1C). A significant positive correlation was revealed between MEgreenyellow module genes and COPD (*r* = 0.57, *p* < 0.001, Fig. 1D). Therefore, genes within the MEgreenyellow module were considered crucial regulators involved in the pathogenesis of COPD. Interestingly, the MEgreenyellow module contained five Rab genes, which were RAB6B, RAB13, RAB32, RAB38, and RAB40B (Fig. 1E).

To validate the expression of these Rabs, RT-qPCR and western blotting were applied on the lung tissue samples, whose clinical data was showed in Fig. 1F. There were seven male and three female patients, with an average age of 70.40 ± 8.17 years old. As one of the diagnosis criteria of COPD, the ratio of forced expiratory volume in one second and forced vital capacity (FEV_1_/FVC) was also listed in Fig. 1F. The mean FEV_1_/FVC value of the non-COPD group was significantly higher than that of the COPD group (Figs. 1G and 82.41% vs. 52.91%, *p* < 0.0001). The relative mRNA expression level of RAB6B, RAB13, RAB32, RAB38, and RAB40B was calculated (Fig. 1H, *n* = 5). The mRNA expression level of RAB32 was higher in the COPD group while no statistical difference was found in the mRNA expression of other Rab genes. The differential expression could also be demonstrated at the protein level (Fig. 1I, *n* = 5).

### Clustering of RAB32 expression patterns in COPD

**Fig. 2:**
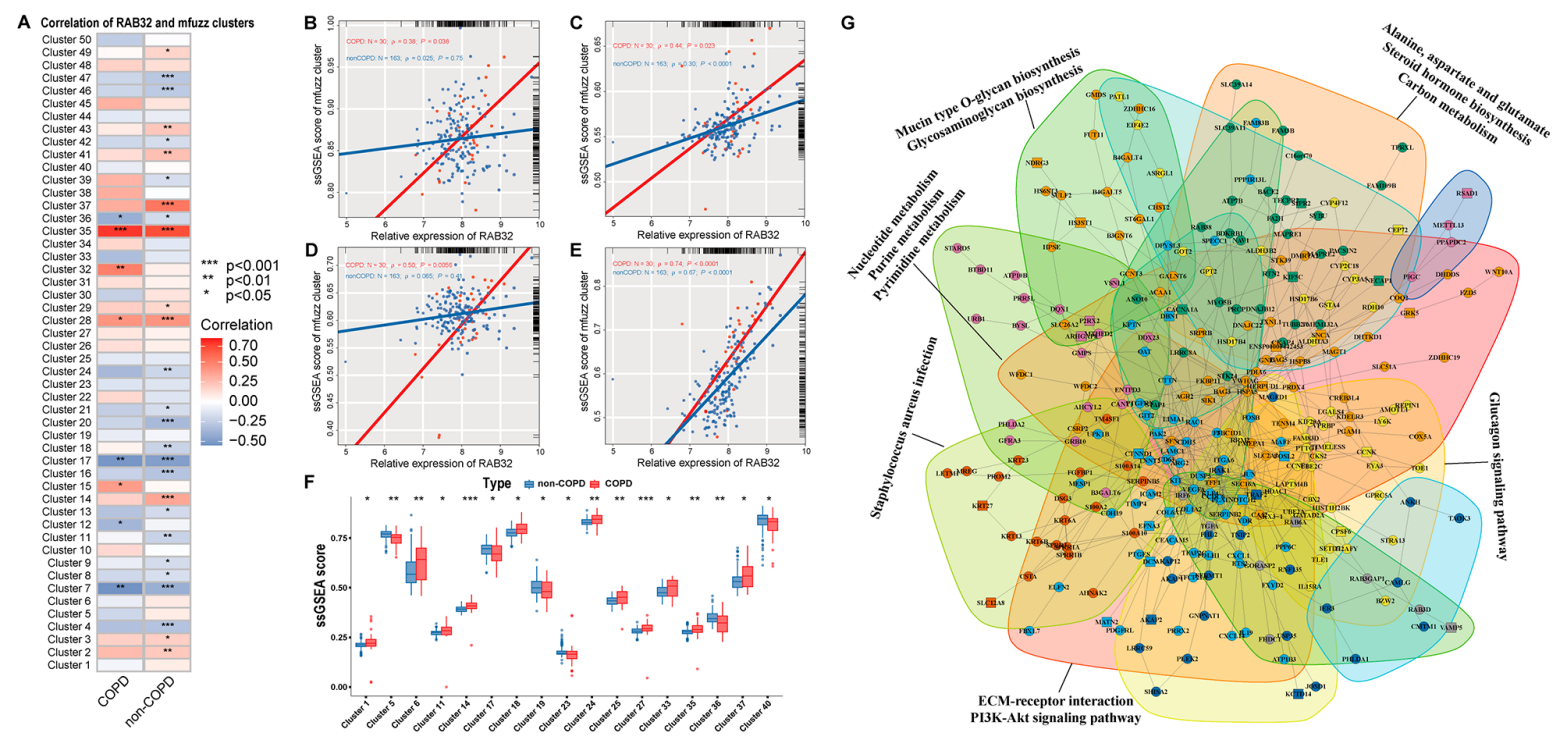
Molecular cluster identification based on RAB32 expression patterns using the Mfuzz algorithm. (**A**) Correlation between 50 Mfuzz gene modules’ ssGSEA scores and RAB32 expression. Each cell displays the respective *P*-value. (**B-E**) Scatter plots showing the correlation between ssGSEA scores and RAB32 expression levels for Cluster15 (**B**), Cluster28 (**C**), Cluster32 (**D**), and Cluster35 (**E**). (**F**) Boxplot displaying the distribution of ssGSEA scores for differentially expressed modules in COPD and non-COPD groups. (**G**) Biological network graph of Mfuzz Cluster35, with different blocks representing neighborhood structures extracted by iGraph network analysis data. The text next to the blocks represents the corresponding biological pathways. Circles of the same color represent participation in the same biological process. Note: *P < 0.05, **P < 0.01, ***P < 0.001, ****P < 0.0001

Genes with similar expression patterns often indicate their involvement in shared biological processes and regulatory mechanisms. Therefore, we employed the Mfuzz algorithm to perform pattern clustering analysis based on the expression levels of RAB32 in both COPD and non-COPD samples. As shown in Fig. S2, Mfuzz identified 50 distinct clusters. The performance of these clusters was evaluated using the ssGSEA algorithm, and the correlation between ssGSEA scores and RAB32 expression levels was analyzed (Fig. 2A). Interestingly, only four modules (Cluster15, Cluster28, Cluster32, Cluster35) exhibited a significant positive correlation with RAB32 expression in COPD (Fig. 2B-E). Surprisingly, Cluster28 and Cluster35 showed significant associations with RAB32 in both COPD and non-COPD samples, albeit with varying degrees of correlation strength (Fig. 2C, E). Additionally, we assessed the differential ssGSEA scores of Mfuzz modules between COPD and non-COPD, revealing that only Cluster35 displayed significantly higher ssGSEA scores in COPD. Based on these findings, we considered Cluster35 to be the most closely linked gene cluster with RAB32.

To further investigate the functional relevance of Cluster35, we performed bioinformatics analysis using the STRING database. This analysis predicted associations between Cluster35 and functionally annotated subnetworks or neighborhoods. Additionally, we utilized the clusterProfiler gene enrichment tool to identify the most important GO terms and KEGG pathways associated with Cluster35 (Fig. 2G). The enrichment analysis revealed that Cluster35 primarily influenced metabolism-related processes, including Nucleotide metabolism, Purine metabolism, Pyrimidine metabolism, Mucin type O-glycan biosynthesis, and Glycosaminoglycan biosynthesis. Furthermore, Cluster35 was found to be involved to a lesser extent in the PI3K-Akt signaling pathway, extracellular matrix receptor interaction, and Glucagon signaling pathway. Notably, Staphylococcus aureus infection was identified as one of the key pathways in which Cluster35 participated, due to its influence on several Keratin-related genes, such as KRT13, KRT23, and KRT27.

### Functional annotation of RAB32 in COPD

**Fig. 3:**
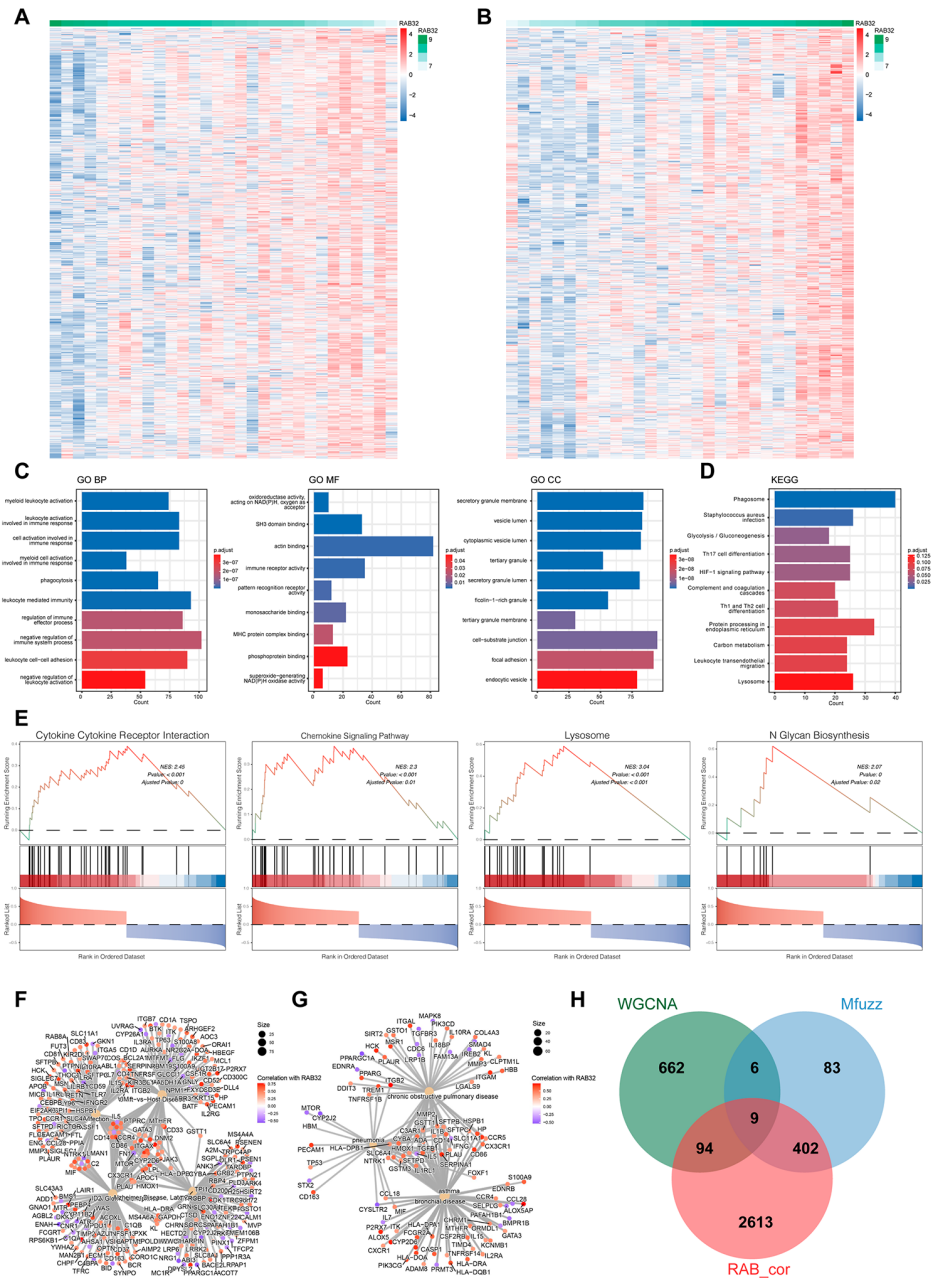
Associated genes of RAB32 in COPD and their involvement in biological characteristics. (**A-B**) Heatmaps showing genes significantly negatively (**A**) or positively (**B**) correlated with RAB32 in COPD patients. (**C**) GO enrichment annotation of RAB32-related genes. (**D**) KEGG pathway enrichment analysis revealing key signaling pathways involved with RAB32. (**E**) GSEA analysis of RAB32. (**F-G**) Enrichment analysis of RAB32 based on the DisGeNET database (**F**) and DO database (**G**). (**H**) Venn diagram illustrating the intersection between WGCNA hub modules, Mfuzz clusters, and RAB32-associated genes

Cluster35 showed a significant correlation with RAB32 in both COPD and non-COPD samples (Fig. 2E), suggesting that the Mfuzz algorithm alone may not fully characterize the potential molecular mechanisms of RAB32 in COPD. Therefore, we conducted a more in-depth investigation using Spearman analysis to identify genes significantly correlated with RAB32 expression in COPD. Among the genes showing overall correlation (FDR < 0.05), 1, 514 genes exhibited a significant positive correlation with RAB32 expression (Fig. 3A, *r* > 0.3), while 1, 604 genes displayed a significant negative correlation (Fig. 3B, *r* < 0.3). Functional enrichment analysis of RAB32-related genes was performed using the Metscape website and clusterProfiler tool. Figure 3C showed the results of the GO analysis, including BP, MF, and CC categories. The results revealed that changes in BP were mainly enriched in negative regulation of the immune system process, followed by leukocyte mediated immunity and leukocyte cell-cell adhesion. In the MF category, significant enrichment terms included actin binding, immune receptor activity, and phosphoprotein binding. Moreover, the CC category was predominantly represented by endocytic vesicle, focal adhesion, cytoplasmic vesicle lumen, and secretory granule membrane. In the KEGG analysis, significantly enriched terms included phagosome, staphylococcus aureus infection, glycolysis / gluconeogenesis, Th17 cell differentiation, and HIF-1 signaling pathway (Fig. 3D). GSEA enrichment analysis based on the overall gene features can provide a more comprehensive understanding of the genes’ impact on biological phenotypes. As shown in Fig. 3E, RAB32 was primarily enriched in cytokine-cytokine receptor interaction, chemokine signaling pathway, lysosome, and N-glycan biosynthesis in COPD.

Additionally, diseases associated with RAB32 were identified using the DisGeNET database and DO database. The enrichment analysis based on the DisGeNET database revealed a relationship between RAB32 and infection (Fig. 3F). The DO analysis found RAB32-related genes mainly enriched in COPD, pneumonia, bronchial disease, and asthma (Fig. 3G). In conclusion, RAB32 played a key role in the molecular regulatory network of COPD and might potentially function through various biological processes in COPD. Integrating the results of WGCNA key modules, Mfuzz pattern clusters, and correlation analysis, a Venn diagram revealed nine potential interacting genes with RAB32, which were CAP1, CRTAP, C20orf24, FAM49B, GPRC5A, HK2, LDHA, PITPNC1, and TWIST2 (Fig. 3H).

### Single-cell expression trajectory of epithelial-derived RAB32 in COPD

**Fig. 4:**
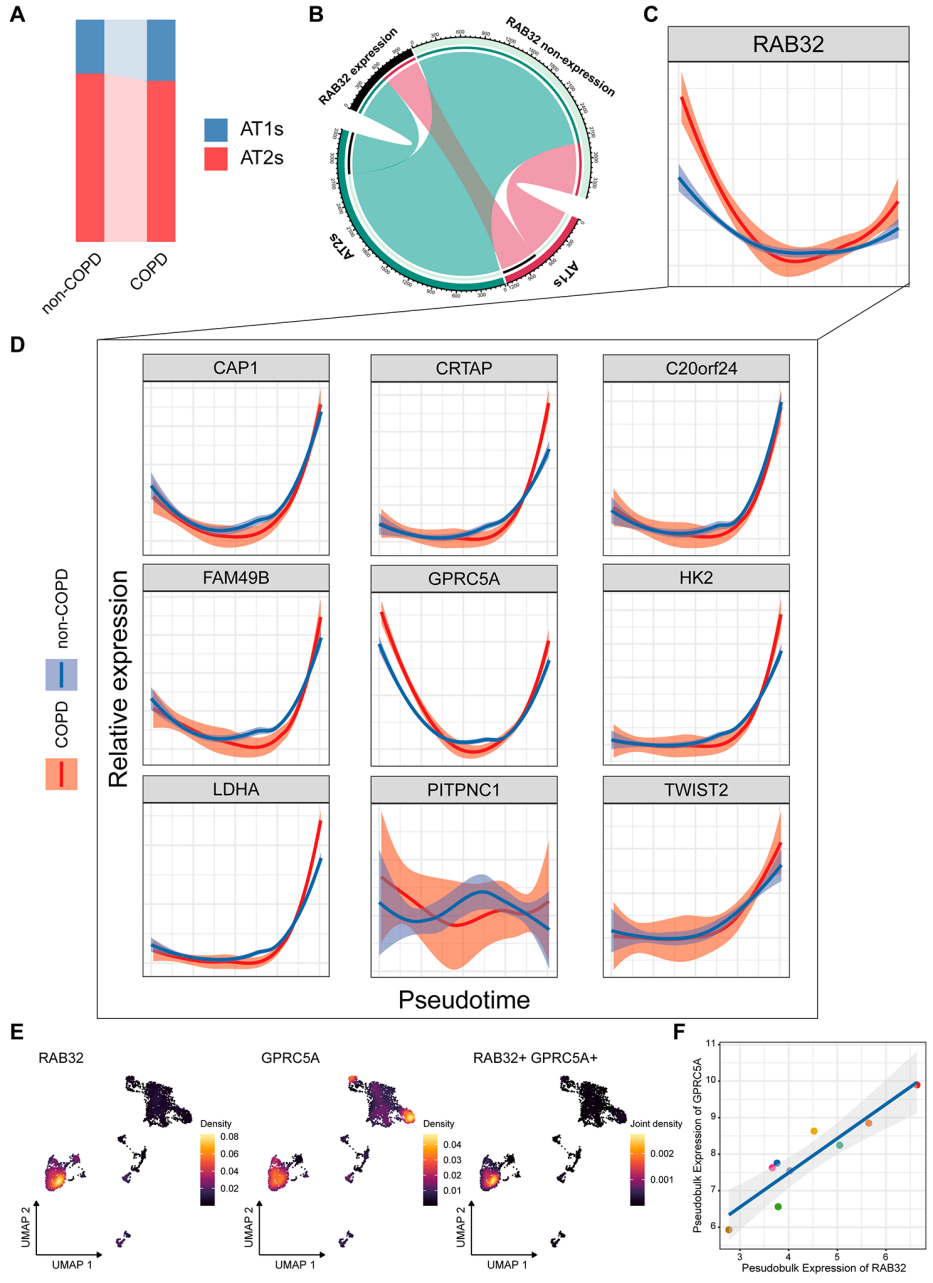
Single-cell analysis revealing expression trajectories and co-expressed molecules of RAB32. (**A**) The relative percentage of AT1s and AT2s in non-COPD and COPD groups. (**B**) Expression of RAB32 in AT1s and AT2s based on origin. (**C-D**) Single-cell expression patterns of RAB32 (**C**) and nine potential interacting molecules (**D**). (**E**) Density plot showing co-expression localization of RAB32 and GPRC5A. (**F**) Scatter plot depicting the correlation between pseudobulk expression of RAB32 and GPRC5A. Note: AT1s, type I alveolar epithelial cells; AT2s, type II alveolar epithelial cells

Previous studies have reported that RAB32 played a role as a cAMP-dependent protein kinase A anchoring protein by interacting with various metabolic organelles in epithelial cells [38]. ScRNA-seq may provide us with a precise perspective to characterize the expression changes of specific genes at the single-cell level. Therefore, we reanalyzed a scRNA dataset (GSE173896) from 12 patients, all of whom provided lung function information to determine their COPD status. After quality control and filtering of individual cells, high-quality transcriptomic data from 4, 771 alveolar epithelial cells (ATs), including 1, 721 cells from non-COPD tissue and 3050 cells from COPD tissue, were extracted (Fig. S3A, B). After removing unwanted cells and merging the data, dimensionality reduction was performed on the 4, 771 ATs for visualization. We annotated and clustered each cell cluster using classical marker genes. The information-rich principal component analysis space was used for UMAP of cell clustering. The composition of ATs in the COPD ecosystem was primarily characterized by AT1s and AT2s (Fig. S4A, B). AT1s showed high expression of AGER, CLIC5, and PDPN, while AT2s displayed high expression of SFTPA1, SFTPA2, SFTPB, and SFTPC. In total, 1, 048 AT1s and 2, 711 AT2s were obtained. We compared the proportions of different ATs between COPD and non-COPD, and it is worth noting that there were slight differences in the preferences for AT1s and AT2s among different groups (Fig. 4A). AT2s predominated in both COPD and non-COPD, while the proportion of AT1s was slightly higher in COPD compared to non-COPD, consistent with previous research results [9, 12].

However, a total of 874 ATs expressed RAB32, with 47.14% localized in AT1s (Fig. 4B). To explore the data distribution characteristics of RAB32 in AT1s, we generated computationally imputed pseudotime trajectories using Slingshot (29,914,354). We observed that the cell branches of ATs followed a linear developmental trajectory and identified the gene expression distribution along the ATs trajectory (Fig. S3C, D). Interestingly, we found that the expression trajectory of RAB32 exhibited a similar distribution between COPD and non-COPD. However, the early expression level of RAB32 was higher in the COPD group compared to the non-COPD group, and it declined and then increased again as pseudotime evolved (Fig. 4C). Surprisingly, among the nine potential interacting genes, the expression trajectory of GPRC5A closely resembled that of RAB32 (Fig. 4D). Co-localization analysis using density plots revealed that RAB32 and GPRC5A were mainly co-expressed in AT1s (Fig. 4E). We also analyzed the correlation between the pseudobulk expression of RAB32 and GPRC5A in different patients and found a significant positive correlation (Fig. 4F, *p* < 0.001).

### Expression of GPRC5A in COPD and co-localization with RAB32

**Fig. 5:**
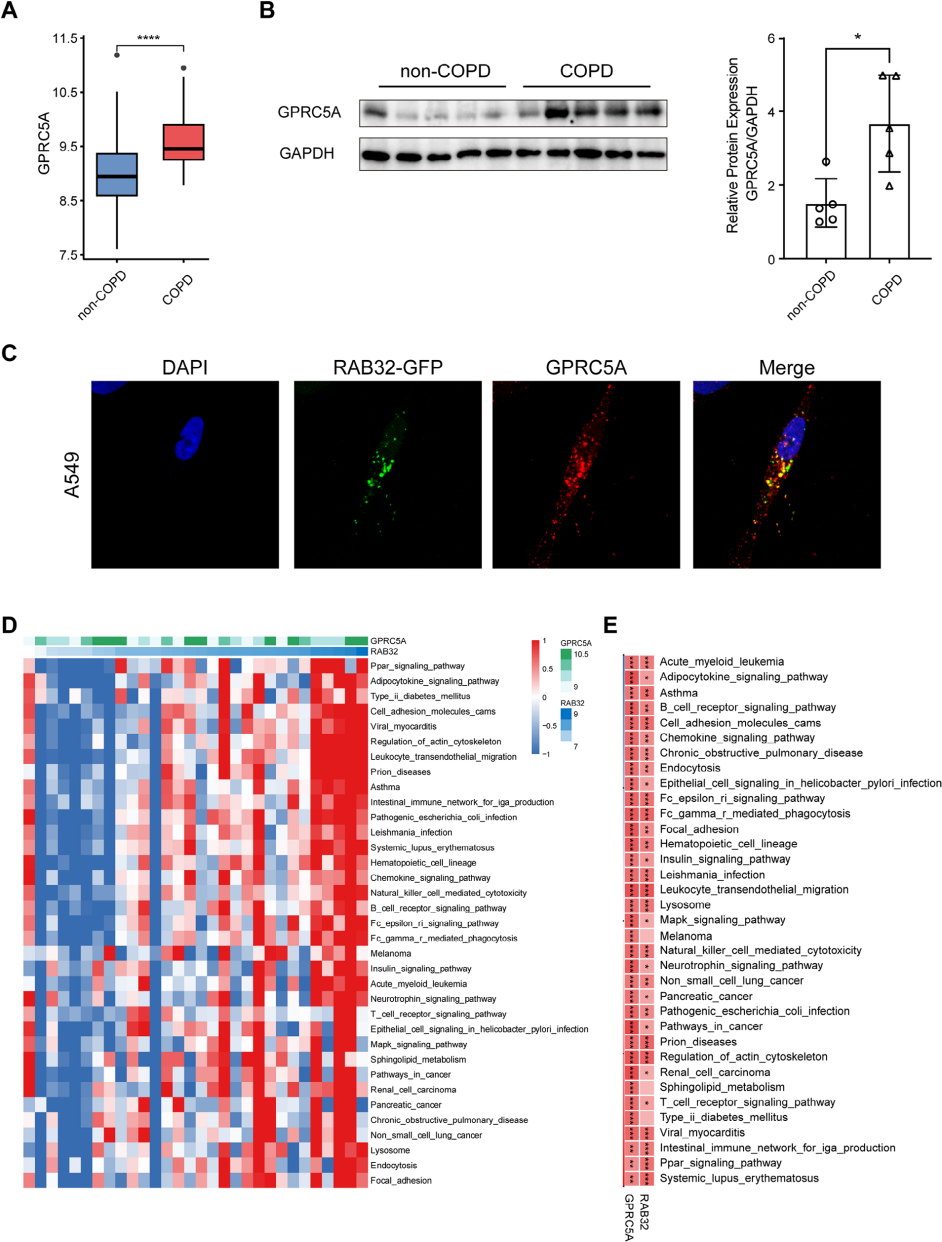
Potential interaction between RAB32 and GPRC5A. (**A**) Differential expression of GPRC5A in non-COPD and COPD samples from mRNA microarray datasets. (**B**) Western blotting and its quantitative results demonstrating protein level differences of GPRC5A between non-COPD and COPD groups. (**C**) Immunofluorescence displaying co-localization of RAB32 and GPRC5A expression in A549 cells. (**D-E**) GSVA analysis of RAB32 and GPRC5A. Note: * *P* < 0.05, ** *P* < 0.01, *** *P* < 0.001, **** *P* < 0.0001

To assess the functional role of GPRC5A in COPD and its potential interaction with RAB32, we performed differential expression analysis of GPRC5A between COPD and non-COPD samples from mRNA microarray datasets. As shown in Fig. 5A, the expression of GPRC5A was significantly upregulated in COPD compared to non-COPD samples. This finding was further validated by conducting western blotting on fresh lung tissue samples of recruited participants. The results revealed a significant increase of GPRC5A expression in the COPD group compared to the non-COPD group (Fig. 5B). Additionally, confocal imaging demonstrated co-localization of GPRC5A and RAB32 in A549 cells (Fig. 5C), suggesting a potential interaction between these two proteins in the context of COPD. To gain insights into the potential mechanisms in which RAB32 and GPRC5A might jointly participate, we employed GSVA. As expected, the expression levels of both RAB32 and GPRC5A were significantly associated with COPD (Fig. 5D, E). Furthermore, we identified several significantly associated pathways, including FcγR-mediated phagocytosis, lysosome, natural killer cell mediated cytotoxicity, regulation of actin cytoskeleton, and cell adhesion molecules. In summary, GPRC5A co-localized with RAB32 in COPD and potentially participated in underlying molecular mechanisms.

### RAB32 and GPRC5A expression in COPD from resected lung tissues

**Fig. 6:**
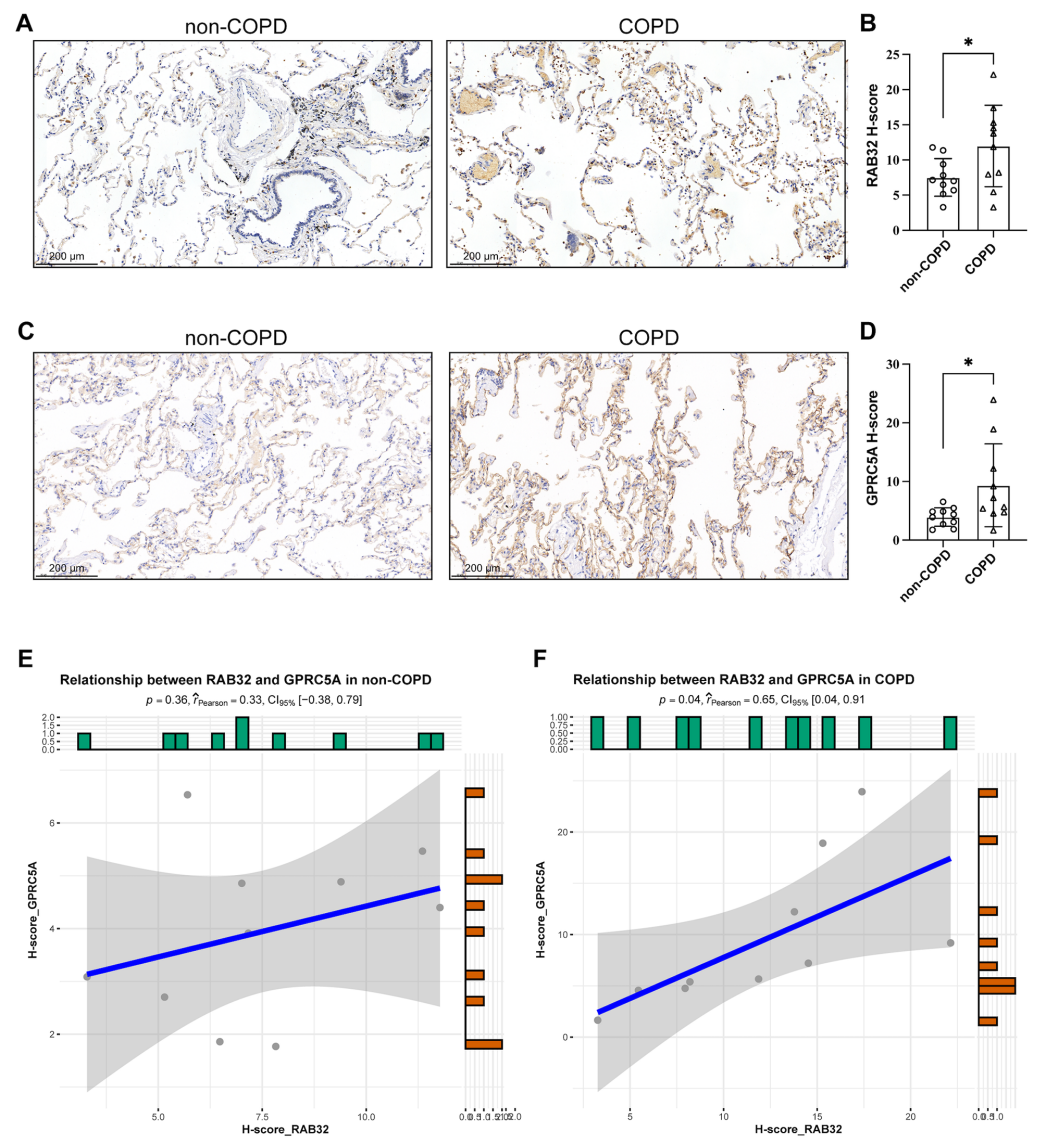
RAB32 and GPRC5A expression in lung tissue samples. (**A**) Immunohistochemistry staining results of RAB32 in lung tissues of non-COPD and COPD patients. (**B**) Bar plot showing RAB32 H-score of non-COPD and COPD patients (*n* = 10). (**C**) Immunohistochemistry staining results of GPRC5A in lung tissues of non-COPD and COPD patients. (**D**) Bar plot showing GPRC5A H-score of non-COPD and COPD patients (*n* = 10). (**E-F**) Pearson correlation analysis of the relationship between RAB32 and GPRC5A in non-COPD patients (**E**) and COPD patients (**F**). Note: * *P* < 0.05, ** *P* < 0.01, *** *P* < 0.001, **** *P* < 0.0001

To visually show the expression of RAB32 and GPRC5A in lung tissues from the COPD and non-COPD group and explore their potential correlation, immunohistochemistry was utilized. Our findings revealed that RAB32 (*p* = 0.046) and GPRC5A (*p* = 0.039) were significantly upregulated in the COPD group compared to the non-COPD group (Fig. 6A-D, *n* = 10). Additionally, we conducted Pearson’s correlation analysis to examine the relationship between RAB32 and GPRC5A in both COPD and non-COPD group. In the COPD group, a notable positive correlation was observed between the expression of RAB32 and GPRC5A. This correlation was found to be significantly higher in the COPD group (*r* = 0.65) when compared to the non-COPD group (*r* = 0.33) (Fig. 6E, F), further corroborating our previous findings.

## Discussion

In this study, we utilized multiple approaches to comprehensively analyze and validate the important role of RAB32 in COPD and identified a potential interacting gene, GPRC5A. WGCNA provided a comprehensive perspective, and the verification experiments on human lung tissues confirmed the critical regulatory role of RAB32 in the pathogenesis of COPD. Mfuzz analysis, based on gene expression patterns in lung tissue, combined with correlation analysis, identified hub genes potentially interacting with RAB32. Furthermore, employing a single-cell perspective, we analyzed the expression characteristics of RAB32 during ATs differentiation in COPD and discovered a robust interacting protein, GPRC5A. Co-localization analysis using confocal imaging and density plots confirmed the co-localization of RAB32 and GPRC5A. In addition, multi-dimensional functional analyses further characterized the potential interaction mechanisms of RAB32 and GPRC5A in COPD.

Rab GTPases are considered crucial members of the vesicular transport machinery, acting as “molecular switches” in processes such as endocytosis, intracellular vesicle trafficking, and cell cytoskeleton dynamics [39–41]. This process is also believed to be involved in the metabolism of epithelial cells, including lipid degradation. Of great interest, both the molecular regulatory network constructed by Mfuzz key clusters and the enrichment analysis of correlated genes identified RAB32’s involvement in metabolic-related processes. Lysosome, an enriched process, was also highlighted. Previous studies have demonstrated the interaction between RAB32 and mTOR kinase: RAB32 depletion regulated the mTOR trafficking to lysosomes and reduced the association of mTOR and mTORC1 pathway proteins with lysosomes [42]. Additionally, RAB32 has been found to direct the ubiquitous machinery for transport from early endosomes to maturing lysosome-related organelles [43]. Therefore, we speculate that RAB32 may exert similar functions in COPD, potentially influencing cellular transport and metabolic functions through lysosomes.

Combining the WGCNA network, Mfuzz co-expression clusters, and correlation analysis, we have identified potential interacting molecules of RAB32. Previous studies have indicated that RAB32 is primarily expressed in epithelial cells. Therefore, we utilized scRNA-seq data of ATs to unravel the expression characteristics of RAB32 in COPD. Our study revealed that RAB32 expression underwent dynamic changes during ATs differentiation in COPD, presenting “a smile curve” with three distinct stages observed. In the early and late stages of differentiation, RAB32 expression was higher in COPD compared to non-COPD, while in the middle stage, RAB32 expression may be higher in non-COPD. Similar reports have previously described this dynamic single-cell gene expression pattern alteration during cellular differentiation [44]. Thus, we speculate that this dynamic change may be related to the disease state or the duration of airway stimulation in COPD. Unfortunately, we were unable to establish specific thresholds for these three stages.

GPRC5A is a G protein-coupled receptor gene [45]. Previous studies have reported decreased expression of GPRC5A in the bronchial epithelium of patients with COPD, and mice lacking GPRC5A were more susceptible to inflammation and lung adenocarcinoma [46, 47]. However, we found a similar dynamic change in the expression pattern of GPRC5A in COPD ATs as observed with RAB32. This suggested that the role of GPRC5A needed to be reassessed, as its expression during different developmental processes might lead to different outcomes. Additionally, we demonstrated through fluorescence confocal analysis and density plots that GPRC5A and RAB32 co-localized primarily in AT1s. Both RAB32 and GPRC5A showed significant enrichment correlations with lysosome, regulation of actin cytoskeleton, and cell adhesion molecules. Therefore, we propose that in alveolar epithelial cells, RAB32 may interact with GPRC5A to regulate lysosomal transport, potentially acting as a novel regulator of cellular metabolism in COPD.

## Conclusions

In conclusion, we have elucidated the expression characteristics of RAB32 in COPD lung tissues. Based on this expression pattern, we conducted a detailed analysis of the potential molecular regulation of RAB32 in COPD. By further integrating single-cell perspective and confocal analysis, we discovered the co-localization of RAB32 with GPRC5A and identified that RAB32/GPRC5A exhibited similar dynamic expression patterns. The RAB32-GPRC5A axis may potentially influence cellular metabolism in COPD by regulating lysosomal transport. Furthermore, we explored the value of the RAB32-GPRC5A axis in clinical samples. Our study provided novel insights into the regulatory mechanisms of RAB32 in COPD, which might serve as a potential target for intervention.

### Electronic supplementary material

Below is the link to the electronic supplementary material.

Supplementary information

## Data Availability

All the data utilized for analysis and processing was described in the method section. On reasonable request, the corresponding author will provide the data and code supporting the conclusions of this work.

## Abbreviations

ATs: Alveolar epithelial cells
BP: Biological processes
CC: Cellular components
COPD: Chronic obstructive pulmonary disease
DisGeNET: Disease Gene Network
DO: Disease Ontology
FDR: False Discovery Rate
FEV_1_: Forced expiratory volume in one second
FVC: Forced vital capacity
GOLD: Global initiative for chronic obstructive lung disease
GEO: Gene Expression Omnibus database
GO: Gene Ontology
GPRC5A: G protein-coupled receptor class C group 5 member A
GSEA: Gene Set Enrichment Analysis
GSVA: Gene Set Variation Analysis
GWAS: Genome-wide association studies
HVGs: Highly variable genes
KEGG: Kyoto Encyclopedia of Genes and Genomes
MF: Molecular functions
PCA: Principal Component Analysis
PPI: Protein-protein interaction
scRNA-seq: Single-cell RNA sequencing
ssGSEA: Single-sample gene set enrichment analysis
TOM: Topological overlap matrix
UMAP: Uniform Manifold Approximation and Projection
WGCNA: Weighted gene co-expression network analysis
