# Supplementary material for: Deciphering the molecular regulatory of RAB32/GPRC5A axis in chronic obstructive pulmonary disease
Source: Respir Res. 2024 Mar 6;25:116. doi: 10.1186/s12931-024-02724-2 (PMC10919015; doi:10.1186/s12931-024-02724-2)
Supplement: Supplementary file 1 — Supplementary information [file 12931_2024_2724_MOESM1_ESM.pdf]

Supplementary Information

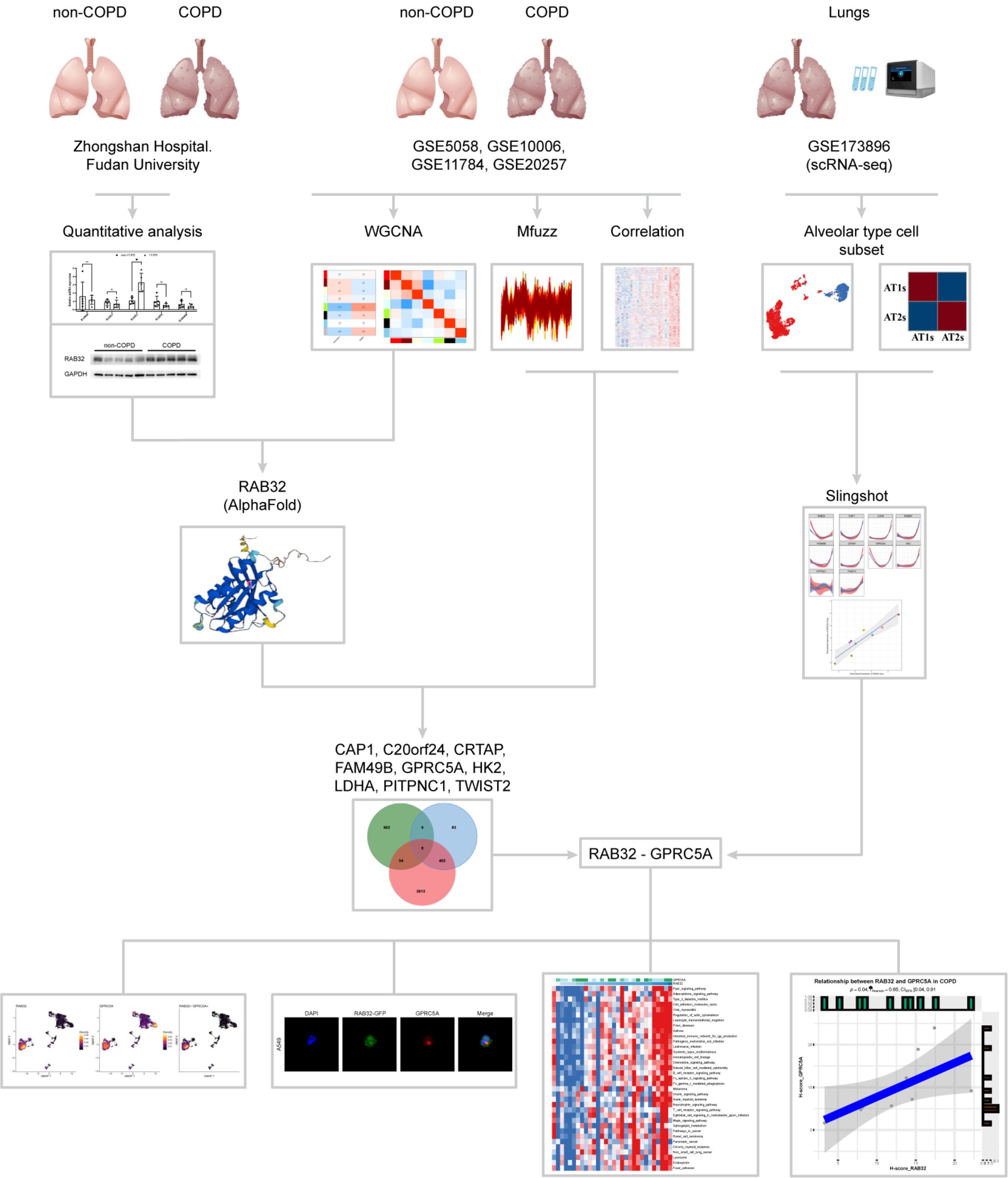

Figure S1. Workflow for experimental design and analysis.

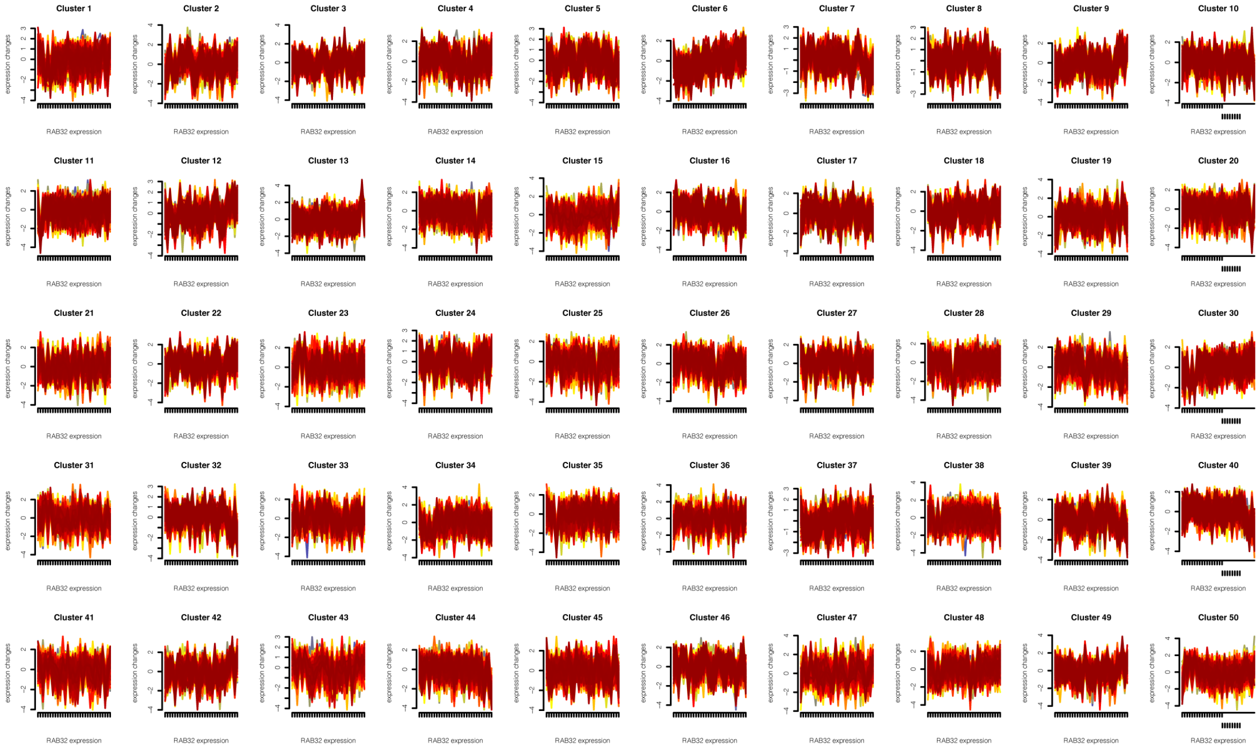

Figure S2. Mfuzz expression pattern clustering results.

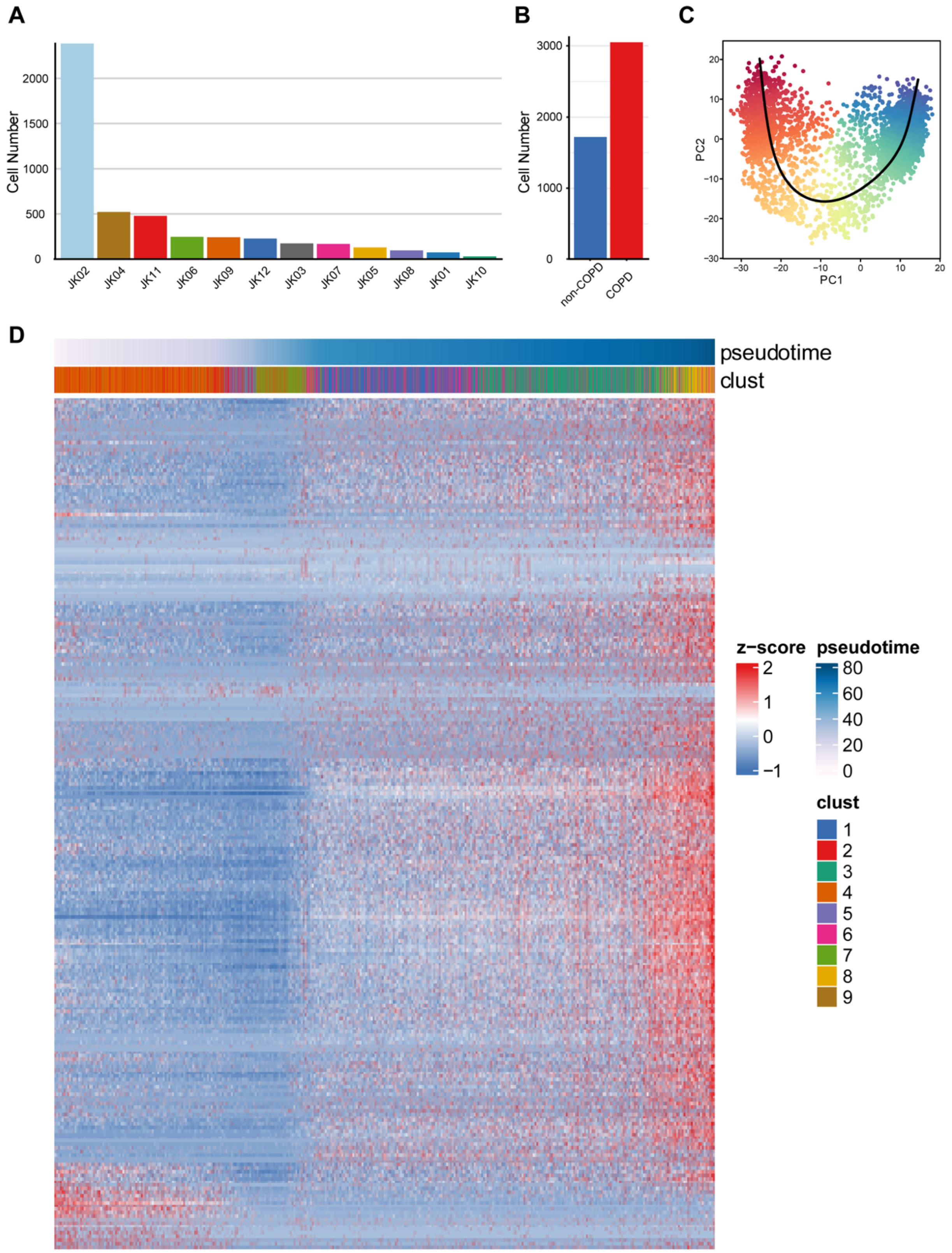

Figure S3. (A) Bar plot showing the number of alveolar epithelial cells included in the analysis for each sample. (B) Number of cells in non-COPD and COPD groups. (C) PCA displaying potential differentiation trajectories of alveolar epithelial cells. (D) Heatmap presenting pseudotime results of alveolar epithelial cells.

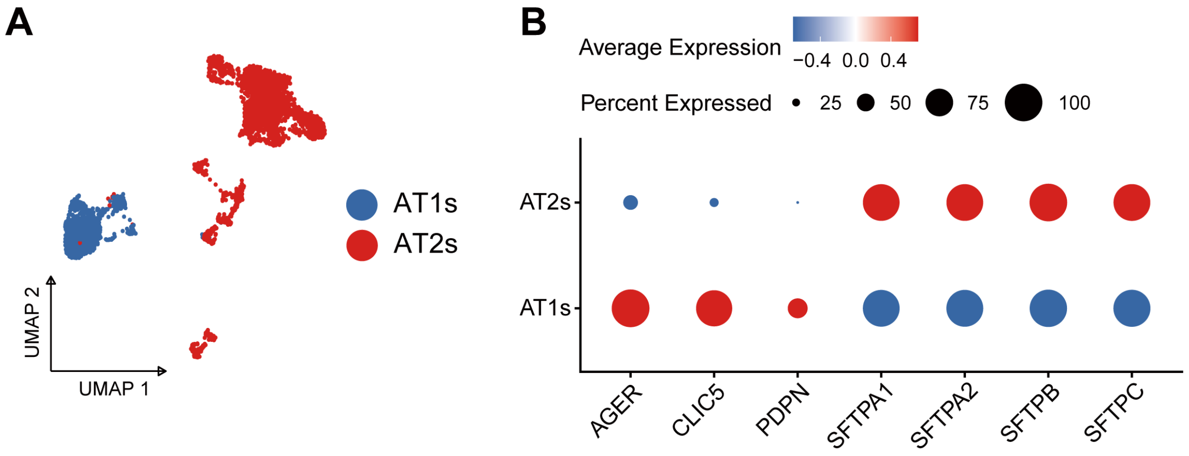

Figure S4. (A) UMAP plot of AT1s and AT2s from single-cell analysis. (B) Bubble plot displaying marker genes for AT1s and AT2s.
